# Land snail dispersal, abundance and diversity on green roofs

**DOI:** 10.1371/journal.pone.0221135

**Published:** 2019-11-14

**Authors:** Michael L. McKinney, Nicholas S. Gladstone, Jillian G. Lentz, Faith A. Jackson

**Affiliations:** 1 Department of Earth and Planetary Sciences, University of Tennessee, Knoxville, TN, United States of America; 2 Department of Zoology, Southern Illinois University, Carbondale, IL, United States of America; Universiti Malaysia Sabah, MALAYSIA

## Abstract

We present the first major systematic study of land snail diversity on green roofs. We surveyed 27 green roofs and the adjacent ground habitat in six major cities in the southeastern United States. We found a total of 18 species of land snails, with three considered to be non-native or invasive species. The majority of land snails encountered in surveys are widespread, generalist species, typically adapted to open habitats. Twelve of the land snails encountered are “greenhouse” species that are very commonly transported via the horticultural trade. Therefore, we infer that at least some land snail species are introduced to green roofs via initial green roof installation and associated landscaping. The major determinants of snail species richness and abundance are the size of each roof and the quality of green roof maintenance regime.

## Introduction

Green roofs (i.e., roofs designed to have substrate and vegetation) are increasingly common in many parts of the world. They are an important part of urban green infrastructure, with many environmental benefits relating to storm water runoff, air and water pollution, urban heat island effects, and improved energy conservation [1]. In addition, green roofs can act as vegetated islands of refugia in an otherwise hostile urban matrix to provide habitat for non-human species and promote overall urban biodiversity [2–3]. Previous studies have documented the role of green roofs as habitat for several groups of organisms, especially mobile groups that can readily colonize them, such as birds [4], bees [5], and other major insect groups [6]. These studies document that many native and non-native species can potentially colonize and persist in green roof habitats. However, studies that document green roof colonization and population persistence of animal groups characterized by low vagility (such as land snails) are quite rare. Only one study incorporating land snail surveys on green roofs was located via literature search, documenting four species found on two roofs in Finland [7].

Along with other invertebrate groups ([8] for review), land snails could be common constituents of green roof environments. Generally, most land snail species in North America are associated with moist forest ecosystems, and the potentially harsh conditions of green roof habitats (e.g., prolonged exposure to direct sunlight, comparatively less shelter habitat) may not support diverse land snail communities. Yet, land snails have been well documented in a variety of urban habitats [9–12], are common “hitchhiker” species that are often transported on commercial materials such as horticultural plants [13] and may even disperse via translocation on larger vertebrate animals [14–15]. Moreover, land snails are often seen crawling on building walls and may actively colonize green roofs on their own.

Here, we conduct the first major and systematic survey of snail occupation of green roof habitat. We surveyed 27 green roofs in six major cities in the southeastern United States. Our objectives were to 1) determine how common and how diverse land snails are on green roofs, 2) investigate the relationships between land snail communities and green roof characteristics, and 3) compare land snail diversity of green roofs to immediately adjacent ground habitats.

## Materials and methods

### Field sites and survey protocol

**Fig 1:**
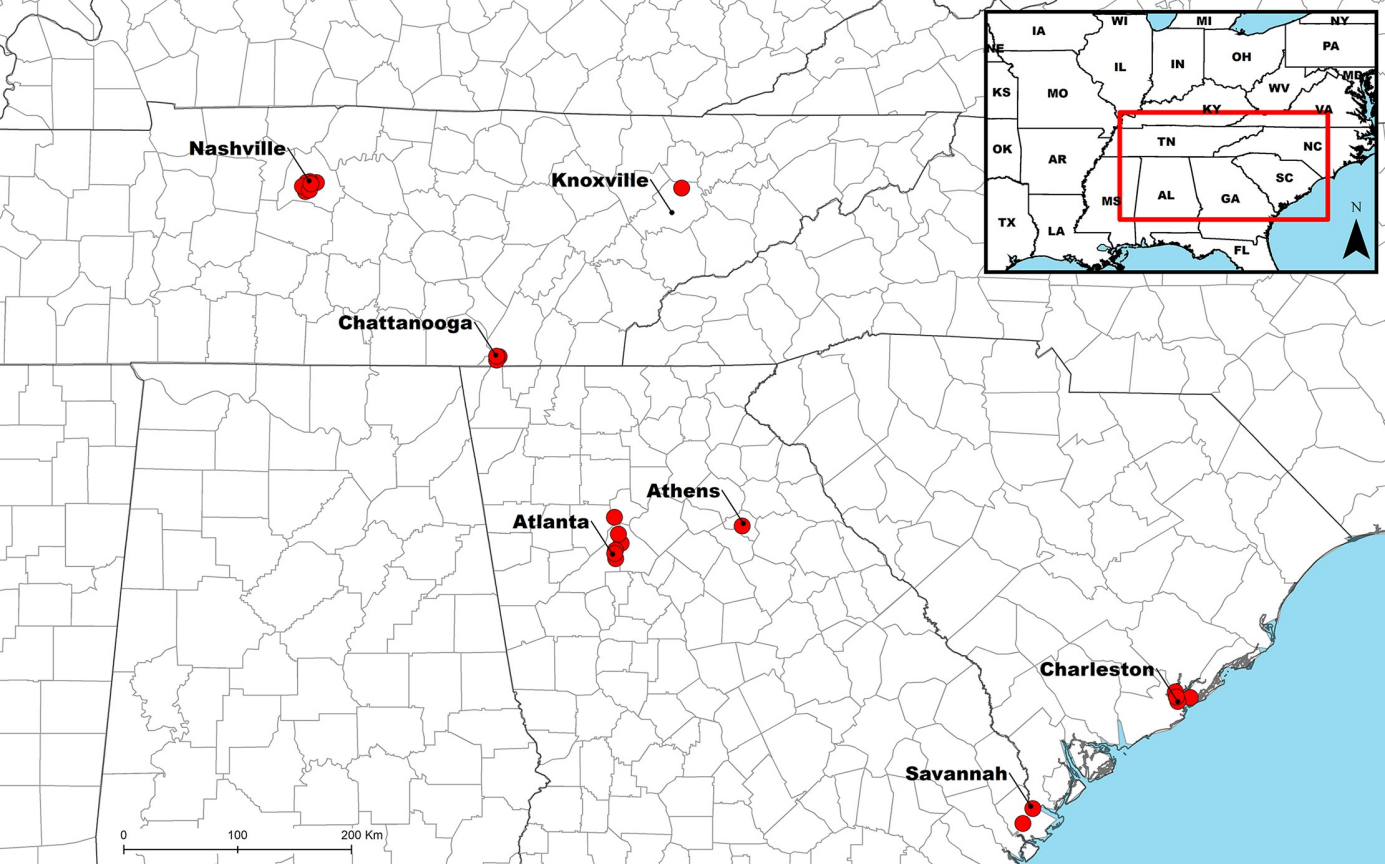
Map of all land snail survey sites with adjacent major cities labeled.

We identified six major metropolitan areas in the southeastern United States that were known to have buildings with designed green roofs. Three of these cities are in Tennessee: Knoxville, Chattanooga, and Nashville. The other three cities were: Atlanta, Georgia; Charleston, South Carolina, and Savannah, Georgia (Fig 1). Buildings with green roofs were located using several methods: internet searches (e.g., www.greenroofs.com), social media, and contacts with green roof design firms in those cities. Each building with a green roof was then contacted to see if we could gain access to perform a land snail survey. To maximize our sampling diversity, we were interested in green roofs of any size and height.

Between March 12, 2018 and November 5, 2018, we visited all identified green roofs that would provide access to our survey team, with a total of 27 sites surveyed. During our visit to each green roof site, we met with facilities managers and collected as much information as possible about each roof: date of greenscape construction, green roof installation company (if any), original plant list and landscaping schedule, and details of regular green roof maintenance (irrigation, weeding, plant replacement, etc.). In cases when building owners did not know the age of each building, this information was procured by means of contacting public records officials for each respective city. In order to determine the sources of the plants encountered, we photographed the identification label of each plant and subsequently researched the associated horticultural facility, whether or not they distributed both local and non-local plants, and whether the plant identified was local or not.

In order to standardize snail sampling effort, we used timed visual searches that were proportional to the area of the roof using the formula of one person searching for 15 minutes per 25 square meters, with no overlap of search areas. During the survey, we collected all dead snail shells that were encountered. Live land snails were photographed in several positions for later identification and then returned to the substrate. While there is often difficulty in identifying land snail shells to species level with the use photographs, we thoroughly photographed as many diagnostic traits as were possible to visualize. Moreover, in nearly all cases, there was dead shell material of the same morphospecies available at each site that were taken back for referenceable identification, and this greatly strengthened the support of the taxonomic identities. Other terrestrial gastropods (i.e., slugs) were not collected during our surveys. Additionally, 0.5 L bags of substrate were collected from each site to search for microsnail species (<5mm in shell diameter; [16]). Substrate types encountered included topsoil that spilled over from potted plants, soil from regularly spaced garden boxes, or an evenly distributed mulch or synthetic growth medium. To standardize substrate sampling effort, one bag of substrate was collected for approximately 25 square meters of roof area. We also collected land snails on the ground around the perimeter of each roof site to examine similarities between roof and ground land snail fauna. We used the same roof sampling procedure for ground collections, with a timed visual search combined with substrate bag samples. Surveys of adjacent ground habitat did not exceed 5 m away from the building.

After the survey, substrate bags were dried for at least two weeks in time before processing. Substrate samples were emptied into a graded series of soil sieves (25.4, 8, 3.36, and 1 millimeters) to extract microsnails. Samples were lightly flushed with water through each sieve grade down to a flat pan to catch all remaining specimens. Sieves were individually inspected following this procedure with a hand lens under direct light to find any shells within each soil sample. All land snails were identified using published keys and species descriptions [17–19], as well as examination by taxonomic specialists (Dan Dourson). All land snail specimens collected were organized by taxonomic identity and location for deposition in the malacology collection of the McClung Museum of Natural History and Culture at the University of Tennessee, Knoxville. In addition to the land snail surveys, we also noted the dominant vegetation and plant diversity of each green roof. In some cases, this was aided by plant lists provided by the building manager or the company that installed the green roof.

For insight into dispersal modes, we compared our green roof land snail fauna to lists of “greenhouse species” that are commonly transported with plants in the horticultural trade [13]. For insight into adaptive potential to green roof habitats, we searched the internet and literature using various sources for information on the natural habitat where each species is typically found [19–20].

### Data analyses

Metrics of community diversity and similarity between both roof and ground habitats were quantified for each site, including species richness, total abundance, Simpson’s diversity index (D), Shannon’s index (H’), Evenness (E), and the Jaccard’s index of similarity. To determine whether these metrics were significantly different between roof and ground habitat, generalized linear mixed models (GLMMs) were applied to scaled data. Habitat type (ground or roof) was utilized as the fixed factor, site as the random factor, and each diversity metric (richness, abundance, D, H’, E) as a response variable. A lognormal distribution best fit all responses, and as such we used a penalized quasi-likelihood approach as implemented in the glmmPQL function of the MASS package [21] in R v.3.6.1 [22]. To assess variation attributable to fixed and random effects within each model, marginal and conditional R^2^ values were calculated using the MuMIn package [23]. To evaluate the effect of green roof habitat variability on land snail species richness and abundance, a separate GLMM was applied using the same procedure to a scaled dataset containing green roof characteristics (roof height, roof area, year of installation, maintenance and plant diversity), with site again serving as the random variable.

## Results

### Survey results and species composition

A total of 27 green roof sites were analyzed, ranging from 5–191,000 sq. ft, from 2–53 years old, and up to 8 stories high (Table 1). Plant diversity on these roofs was roughly evenly distributed between low, medium and high, with *Sedum* being the dominant vegetation on many roofs (S1 Table) as is commonly the case with green roofs due to their drought tolerance. Land snail species richness ranged from zero to nine species, with seven sites having no species observed and only five surveyed sites containing more than three species.

**Table 1:** Summary of green roof survey results ordered by snail richness.

Green Roof Building	City	Area (ft^2^)	Maintenance	Height	Age (yrs)	Plant Diversity	Snail Richness
Freeman Webb Building	Nashville	52272	High	5	9	Moderate	9
Creative Museum	Chattanooga	3802	High	3	8	High	8
Shelby Bottoms	Nashville	3200	High	2	10	High	7
McCabe Community Center	Nashville	2750	High	2	7	Moderate	6
Mary Walker	Chattanooga	2200	Moderate	2	7	Moderate	4
Atlanta Botanical Gardens	Atlanta	4500	High	3	9	High	3
Homewood Suites- 8th Floor	Savannah	1000	Moderate	8	3	Moderate	3
Low Country Local First	Charleston	2300	Moderate	2	4	Moderate	3
Sevier Park Community Center	Nashville	2000	Moderate	2	4	Moderate	3
University of GA, Geog/ Geol Bldg.	Athens	3000	High	3	53	High	3
Atlanta Botanical Gardens (Side Bldg.)	Atlanta	500	High	2	9	High	2
Cal Turner Family Center	Nashville	14600	Low	2	4	Moderate	2
Clemson University Restoration Institute	Charleston	2560	Low	3	2	Low	2
Georgia Southern Uni, Learning Commons	Savannah	500	Low	2	5	Moderate	2
Southface Energy Institute	Atlanta	2000	Low	2	10	Low	2
Chattahoochee Nature Center	Atlanta	2000	Low	2	9	Low	1
Homewood Suites- 3rd Floor	Savannah	1000	Moderate	3	3	Moderate	1
Mt. Pleasant MUSC East Cooper Hospital	Charleston	700	Low	3	7	Low	1
Music City Center	Nashville	191000	High	6	6	Low	1
Zoo Atlanta	Atlanta	16000	High	2	22	High	1
ASHRAE Headquarters	Atlanta	1800	Low	2	10	Low	0
Crash Pad	Chattanooga	3000	Low	2	6	Moderate	0
First TN Park	Nashville	1500	Low	2	3	Low	0
Gibbs High School	Knoxville	200	Low	1	8	Low	0
Hamilton County Health Center	Chattanooga	5400	Low	2	7	Moderate	0
Saul Nurseries "The Swamp"	Atlanta	50	Low	1	16	Low	0
Sky Garden	Charleston	500	Low	2	8	Low	0

**Fig 2:**
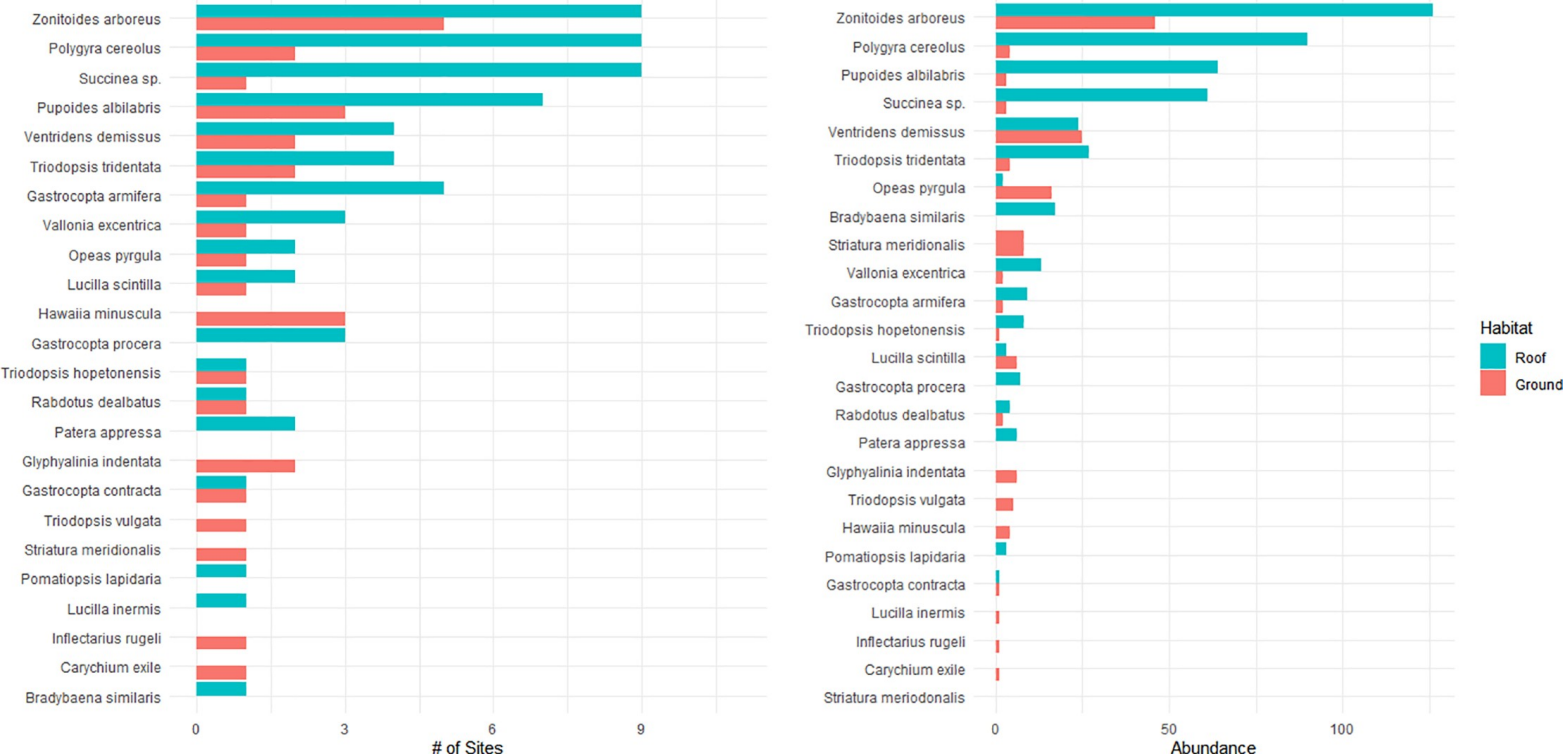
Snail diversity summarizing number of green roof sites found and total abundance.

A total of 18 land snail species were found on the 27 green roofs with the taxa found on the most green roof sites being: *Succinea sp*., *Zonitoides arboreus*, *Polygyra cereolus*,and *Pupoides albilabis* (Fig 2), each being found on seven or more sites. These four species were also the most abundant with a total of 61, 126, 90 and 64 individuals of each being found, respectively (Table 2). Thus, these four species account for 73.2% of all land snails encountered on green roofs. A substantial number of snails found showed evidence of a living population: 44 of 67 occurrences (67.7%) contained at least some snails that were recently dead (with tissue) or still alive (S1 Table).

**Table 2:** Summary of green roof and ground-dwelling species. “Greenhouse” designation in relation to survey results from [13]. Habitat information was obtained from [19–20].

	Sites	n	Greenhouse	Habitat
**Roof Species**				
*Succinea sp*.	9	61	Y	Amphibious, wet leaf litter
*Zonitoides arboreus*	9	126	Y	Generalist, v. widespread
Polygyra cereolus**	8	90	Y	Coastal, disturbed areas, gardens
*Pupoides albilabris*	7	64	Y	Glades, urban, bare ground
*Gastrocopta armifera*	5	9	Y	Sunny, open areas, urban
*Triodopsis tridentata*	5	27		Generalist, disturbed urban
*Ventridens demissus*	4	24	Y	Generalist, leaf litter, urban
*Gastrocopta procera*	3	7	Y	Dry open ground
*Vallonia excentrica*	3	13		Grassy meadows, urban
*Lucilla scintilla*	2	3		Open grassy areas
Opeas pyrgula*	2	2	Y	Open disturbed urban
*Patera appressa*	2	6	Y	Open disturbed urban
Bradybaena similaris*	1	17	Y	Generalist, grassy, humid
*Gastrocopta contracta*	1	1	Y	Generalist, many habitats
*Lucilla inermis*	1	1		Open grassy areas
*Pomatiopsis lapidaria*	1	3		Moist soils, wetlands
*Rabdotus dealbatus*	1	4		Open glades, meadows
*Triodopsis hopetonensis*	1	8	Y	Generalist, disturbed urban
**Ground Species**				
*Zonitoides arboreus*	5	44	Y	Generalist, v. widespread
*Hawaiia miniscula*	3	4	Y	Bare ground, urban
*Pupoides albilabris*	3	3	Y	Glades, urban, bare ground
*Glyphyalinia indentata*	2	6	Y	Generalist, leaf litter, urban
Polygyra cereolus**	2	4	Y	Coastal, disturbed areas, gardens
*Triodopsis tridentata*	2	4		Generalist, disturbed urban
*Ventridens demissus*	2	25	Y	Generalist, leaf litter, urban
*Carychium exile*	1	1		Dense leaf litter, talus slopes
*Gastrocopta armifera*	1	2	Y	Sunny, open areas, urban
*Gastrocopta contracta*	1	1	Y	Generalist, many habitats
*Inflectarius rugeli*	1	1		leaf litter, upland woods
*Lucilla scintilla*	1	6		Open grassy areas
*Striatura meridionalis*	1	8		Meadow, open, urban
*Opeas pyrgula*	1	16	Y	Open disturbed urban
*Rabdotus dealbatus*	1	2		Open glades, meadows
*Succinea sp*.	1	3	Y	Amphibious, wet leaf litter
*Triodopsis hopetenensis*	1	1	Y	Generalist, disturbed urban
*Triodopsis vulgata*	1	5		Generalist, disturbed urban
*Vallonia excentrica*	1	2		Grassy meadows, urban

* = exotic

** = extralimital.

Interestingly, twelve of the 18 green roof land snail species are common “hitchhiker” species (Table 2) found in greenhouses which serve as the transport hubs of horticultural plant species [13]. The general ecological adaptations and habitat preferences of the 18 species of green roof land snails are similar, with most, if not all of them, being generalists adapted to open, grassy and/or disturbed anthropogenic habitats [17, 19]. However, as discussed below there are differences in the maintenance regimes of these 27 roofs that affect their habitability and snail fauna.

Only three of the 18 green roof species are not native to their locations. We found two exotic species (*Opeas pyrgula*, *Bradybaena similaris*) native to southeastern Asia and one known extralimital species to all sites for which it was discovered (*Polygyra cereolus*). We note, however, that the native distribution of *P*. *cereolus* may include the sites within Charleston, SC and Savannah, GA, although these locations are still northward of previously recorded occurrences [20]. The most widespread and abundant of these three species was *P*. *cereolus*, with 90 individuals found at eight green roofs in five cities. *O*. *pyrgula* was found at two green roofs in two cities (one individual each) and *B*. *similaris* had 17 individuals at only one location. In addition to these three species, it is possible that at least some of the succinids are not native but this is uncertain due to the difficulty in differentiating species in this taxonomically challenging group [13].

On the ground habitat adjacent to the green roofs, we found a total of 19 species (Table 2). Two of the most abundant species (*Zonitoides arboreus*, *Pupoides albilabis*) were also among the most abundant on green roofs. Six (31.6%) of these ground species were not found in the pool of green roof species. Ten (52.6%) of ground species are listed as hitchhiker, greenhouse species compared to twelve (66.7%) of roof species. We found the same species of non-natives on the ground as on the green roofs (*Opeas pyrgula*, *Polygyra cereolus*) apart from *Bradybaena similaris* which was absent from all ground samples.

### Data analyses

GLMM results show a significant difference between green roof and ground habitats in all diversity metrics (Table 3). Site location accounted for the majority of variation in the dataset, indicating that individual roof and ground diversity are likely location dependent. In most cases, we were not able to generate a value for the Jaccard Index, as there were many sites with no land snails on the roof, on the ground, or both. However, the three sites with the highest roof species richness (Freeman Webb Building, Creative Museum, Shelby Bottoms) had a Jaccard Index of 0.385, 0.125, and 0.125 respectively. The highest overall Jaccard Index of all sites was 0.5 at the McCabe Community Center with six species on both the roof and ground habitat of which three species were shared. These results indicate that, even in instances of having viable roof and ground habitat for land snails, species composition is often not similar between the two habitats.

**Table 3:** GLMM results indicating significant difference in all diversity metrics between ground and roof habitats.

Response	Estimate	Std. Error	t-value	*R*^2^m	*R*^2^c	*P*-value
Richness	0.346	0.115	2.996	0.014	0.151	0.0061*
Abundance	1.719	0.361	4.756	0.007	0.014	0.0001*
*D*	0.169	0.063	2.717	0.063	0.240	0.0118*
H'	0.182	0.065	2.794	0.043	0.439	0.0098*
E	0.149	0.055	2.695	0.054	0.372	0.0124*

*significant p-value

Additionally, our subsequent GLMM results show that the roof area and maintenance regime all have significant effects on species richness and abundance (Table 4).

**Table 4:** GLMM results indicating a significant effect of roof area and maintenance regime on species richness and abundance.

	Estimate	Std. Error	t-value	*P*-value
*Species Richness*				
Height	0.0639	0.083	0.771	0.449
Age	-0.016	0.011	-1.407	0.174
Area	7.6e-6	3.6e-6	-2.112	0.047*
Plant Diversity	-0.145	0.201	-0.722	0.478
Maintenance	0.762	0.184	4.132	0.0005*
*Abundance*				
Height	0.075	0.149	0.506	0.618
Age	-0.032	0.021	-1.515	0.145
Area	2.01e-5	9.5e-6	-2.123	0.046*
Plant Diversity	-0.588	0.531	-1.109	0.280
Maintenance	1.086	-.463	2.346	0.029*

*significant p-value

## Discussion

### Snail diversity and environmental influences

We found a considerable abundance (466) and species richness (18) of land snails on green roofs. However, it is notable that over one-fourth (25.9%) of the green roofs had no land snails. These snail-free green roofs were all categorized as low-maintenance, including some green roofs that had experienced no maintenance (watering, weeding, replanting) for years (e.g., Gibbs High School). The green roofs that often exhibited high species richness (with three or more land snail species) and abundance all had moderate to high maintenance regimes, and were generally larger in total roof area. These observations, coupled with the results of our data analyses, show that increased land snail species richness and abundance are impacted by increased green roof maintenance and the overall size of each green roof. Moreover, we note that although plant diversity was not identified as a significant predictor for either richness or abundance, larger and better maintained green roofs were associated with an overall higher vegetation cover (a field measure that was not directly recorded during this study). This makes sense given that some of the major habitat variables that promote land snail diversity and abundance are moisture, vegetative cover and coarse woody debris [12, 24–25]. A small, completely neglected green roof with little or no watering during drought periods and sparse vegetation will likely be poor habitat for land snails, especially in the hot summer months of the southeastern U.S. While we did not test the pH of growing medium substrates on these roofs, it is not likely to be a major influence in our findings as the mineral-rich nature of commercially used substrates in most green roofs have generally basic (>7) pH values [26].

Despite the overall significance of roof area and maintenance in the promotion of species richness and abundance, we also found that land snail diversity and abundance were at times best explained by somewhat idiosyncratic local environmental conditions. This dominant effect of local environmental conditions on diversity has also been found in green roof studies of other taxa such as beetles, where local habitat traits have a stronger effect on community composition than landscape variables [27]. Moreover, this local effect seems to be especially strong for low-mobility invertebrates [28]. An example of this location-dependency in our data is the green roof at Zoo Atlanta–one of the oldest (22 years), largest, highly vegetated, and well-maintained sites. Despite these characteristics, only one snail species was discovered–a very common ground-dwelling species at the zoo (*Ventridens demissus*). Because of this anomalous finding, a second roof survey was done at this location and confirmed this low diversity. Follow-up discussions with the Zoo Atlanta green roof management indicated that this anomalously low snail diversity may be attributable to the fact that no new horticultural plants have been added to the roof since the green roof installation 22 years ago. If horticultural transport is a major source of dispersal, as noted below, then it may be that snail populations on green roofs may become extirpated if not regularly replaced.

### Dispersal to roofs

Most studies of green roof faunal colonization look at highly mobile taxa such as flying insects which can readily colonize green roof habitat via their own locomotion [2, 8]. For organisms characterized by low vagility, an alternative mechanism of dispersal (i.e., translocation via landscaping and horticulture) may be the most influential for colonizing green roofs. It is well documented that plant nurseries are hot spots for many land snail species and that horticultural plants are major mechanisms of land snail introductions [13, 29]. In this study, we find that most land snail species encountered are indeed well-documented greenhouse inhabitants (Table 2). A similar mechanism has been suggested for the dispersal of Collembola (springtails) onto green roofs via composting for roof soil enrichment [30]. If our findings are validated with further research, it could provide useful insight into the dynamics of how green roof ecosystems are created and change through time. It also indicates that humans can have some control over which land snails colonize these designed ecosystems.

Our findings do not directly suggest a role for self-dispersal via land snail locomotion. Many ground habitats adjacent to our green roof sites are mainly “hardscapes” with little or no vegetation. This is reflected in our survey data which often found no ground snails (S2 Table), and thus reduces our sample size to make roof-ground comparisons. When land snails were found in both ground and roof habitats at a site, there was generally little similarity between the species presence as indicated by the low Jaccard’s Index for each site. Snail locomotion is famously slow. For example, [31] recently documented that the spread of the non-native *Cornu aspersum* across 16 residential yards in a single city block in Norman, Oklahoma took six years. Though land snails have the common tendency to crawl on the (often calcium-rich) walls of some younger buildings, neither age of the building or height of the green roof had a significant effect on species richness or abundance. The two most species-rich roofs (Freeman Webb and McCabe Community Center) and the nearby ground habitats of these sites share several species that may imply active land snail dispersal and interchange between ground and roof communities. However, given that both the roof and ground properties are owned by the same respective entities, the associated landscaping and plantings for both are probably similar. This said, the similarity of roof and ground fauna is more likely a function of plant hitchhiking and landscaping practices.

### Comparison to other green roof fauna

As has been found with several other invertebrate taxa [2, 28, 32], the species inhabiting green roofs tend to be widespread, generalist and disturbance-adapted species. The same studies also show that just a few species tend to dominate in terms of total abundance and being the most widespread among green roof habitats. This is true in our study where just four species account for almost three-fourths of all snails found. Also as found in many other invertebrate studies [5, 32], most of our green roof species are native, with a minority (at least three species) being invasive, broadly adapted non-native species.

In terms of species richness, our findings indicate that land snails on green roofs may be considerably less diverse than more mobile taxa. For example, [5] surveyed just nine green roofs in Vienna, Austria, and identified 90 wild bee species. A study of 17 green roofs in Switzerland found 161 highly dispersive beetle species [28].

### Conclusions and practical applications

In their review of invertebrates on green roofs, [8] point out that “it is not clear whether they adequately provide habitat or not”. They also note that green roof habitats can vary widely, with some green roofs providing very little habitat for invertebrates. Their relative isolation in a hostile urban matrix prevents colonization and this often combines with the harsh conditions on roof tops to inhibit long-term population persistence. Therefore, we need many studies of all major invertebrate groups to truly understand the extent that green roofs can promote invertebrate biodiversity in urban areas. While several invertebrate taxa have seen a growing literature on green roof habitats, here we provide the first extensive study of green roof habitat for land snails.

Our study indicates that large, well maintained green roofs can support a modest land snail community of mostly native taxa. Moreover, our findings further support the importance of local environmental controls on diversity and community composition in green roof ecosystems. Factors such as building height, age, and plant diversity are generally less important determinants of species richness and abundance than size and habitat quality, which can vary widely with vegetation management practices. Furthermore, as with many other taxa groups, the introduction of land snails to green roof habitats is most likely driven by human introduction via the transport of cultivated plants rather than active dispersal.

## Supporting information

S1 Table Summary of all data associated with green roof surveys. (XLSX)

S2 Table Diversity metrics for each survey site. (XLSX)
